# Exploring the Effect of Social Support and Empathy on User Engagement in Online Mental Health Communities

**DOI:** 10.3390/ijerph18136855

**Published:** 2021-06-26

**Authors:** Yixin Chen, Yang Xu

**Affiliations:** Department of Information Management, Peking University, Beijing 100871, China; chenyix@pku.edu.cn

**Keywords:** online mental health community, social support, empathy, reddit, user engagement

## Abstract

It is known that social support and empathy are beneficial for mental health. As a result of the widespread development of social media, online social support and empathy could also influence user behaviors during the development of online communities. However, few studies have examined these effects from the perspective of online mental health communities. These communities appear to be a crucial source for mental health related support, but the spread of online empathy in these communities is not well-understood. This study focused on 22 mental health related subreddits, and matched and compared users (1) who received social support with those who did not receive social support, and users (2) who received more empathic social support with those who received less empathic social support. The results showed that social support and empathy are “contagious”. That is, users who received social support at their first post would be more likely to post again and provide support for others; in addition, users who received more empathic support would subsequently express a higher level of empathy to others in the future. Our findings indicate the potential chain reaction of social support and empathy in online mental health communities. Our study also provides insights into how online mental health communities might better assist people to deliver social support that can help others to deal with mental problems.

## 1. Introduction

It is known that social support can positively affect psychological well-being [1] and alleviate mental health problems, e.g., depression [2,3,4], stress [5], and mental disorder [6]. Research has explored the mechanism in which social support works [7,8] and demonstrated the impact of social support on different populations, such as college students [9], elderly people [10], and low-income populations [11]. With the rapid development of social media, online mental health communities (OMHCs) are widely used for mental health purposes [12,13,14] and are regarded as an important channel to understand people’s mental states [15,16]. Considering the basic structure of OMHCs, research views the posts on mental health communities as seeking support and the comments as providing support [17,18].

Users play the role of not only recipients but also providers of social support in online communities. Their willingness to provide social support is essential for the development and success of online communities [19,20]. Therefore, in addition to the impact of social support on mental health, studies also explored its relationship with user engagement and community development. Interviews of online users suggested that social support boosted subsequent feelings of pride, self-confidence, and status, and therefore motivated content contribution [21]. Studies on some specific online communities such as weight-loss communities also showed that social support could facilitate users’ following engagement, such as increasing return probability [22,23].

In the process of providing social support or commenting in OMHCs, users could also express empathy to others they respond to. In this context, empathy exists in the text-based form instead of the face-to-face or speech-based form that most research on empathy focused on. Similar to traditional empathy, online empathy is also proven to be related to a positive outcome, such as willingness to content contribution [24] and interpersonal interactions [25]. Considering the potential positive effects of online empathy on community development and user experience, recent studies have proposed ways to measure or even improve empathy in online responses or comments [25,26,27,28].

Although prior work has shown the positive effects of social support or empathy on people’s mental health state and user contributions, few studies examined these effects from the perspective of online mental health communities, which is a crucial source for online mental health support [29]. In addition, few studies further investigated the empathy level of online social support instead of focusing on the general forms of social support such as the number of comments. Furthermore, even though the association between offline social support and empathy level has been explored before [30], it is not clear how social support or empathy is related to users’ subsequent expression of empathy in the online context. Therefore, our work seeks to address the research gap by focusing on OMHCs in Reddit and examining the impact of received social support and empathy on individuals’ subsequent behaviors.

In this study, we conducted propensity score matching to match users who received social support with those who did not receive social support at their first post, and compared their probability of returning to OMHCs as well as their subsequent empathy expression. We also calculated the empathy level of their received comments and used a similar approach to analyze the impact of average empathy level of received comments on users’ subsequent behaviors. This study suggests the “contagious” feature of social support and online empathy: users who received social support on their first post would be more likely to return to OMHCs to post again or provide support for others; users who received more empathic support would not only be more likely to return to OMHCs, but also express a higher level of empathy in the future. We also conducted a preliminary content analysis to explore the difference between posts that received the most comments and the least comments, and those received the most and least empathic comments.

This study contributes to the big picture of social support and empathy in an online context and has implications for a better understanding of the social dynamics underlying online mental health communities. Different from previous research on the psychological effects of social support and empathy, our research reveals their significance to user engagement. The contagious effects of social support and empathy found in this study also provide insights into the design of online mental health communities. For example, platforms could help boost the potential chain reactions of social support and empathy to facilitate the spread of them in OMHCs and achieve a sustained and sound development.

## 2. Related Work

### 2.1. Social Support in Online Mental Health Communities

Social support is viewed as “the clarity or certainty with which an individual experiences being loved, valued, and able to count on others should the need arise” [31]. Cutrona and Suhr [32] developed the Social Support Behavioral Code which includes five nuanced types of support-intended communication behaviors: informational support, instrumental or tangible support, emotional support, network support, and esteem support.

Prior research has analyzed the effect of social support on mental health problems, such as depression [2], stress [5], and willingness to quit smoking [33]. Winzelberg et al. [3] randomly assigned 72 women with breast carcinoma to web-based social support group and control group, and the results showed that social support is useful in reducing depression and cancer-related trauma. Lieberman et al. [4] also conducted experiments on people with breast carcinoma and demonstrated that social support significantly reduced depression and reactions to pain. Dalgard et al. [6] conducted a ten-year follow-up study which showed that social support reduced the risk of developing mental disorder when being exposed to negative events and therefore exerted a positive effect on mental health. In addition to the effect on people who receive support, research also showed that getting involved in social support is beneficial for support providers themselves [34,35].

With the development of social media, online communities eliminate geographical barriers [36] and become increasingly popular among people with mental health concerns [37]. As there is limited access to receive offline support [38] and prevalent stigma about mental health problems offline [39], people with mental health concerns may turn to OMHCs to seek support. Pew Research Center [14] reported that in 2012 among online health information seekers, 16% tried to find others who might share the same health concerns and 26% of Internet users have read or watched someone else’s experience about health or medical issues. Oh et al. [40] surveyed 291 Facebook users and the result showed a positive relationship between having health concerns and seeking health-related social support. Utz and Breuer [41] conducted longitudinal studies and suggested that individuals with lower well-being are more likely to turn to social networks for social support. OMHCs enable self-disclosure about mental health issues [12], and the anonymity of some OMHCs makes individuals more willing to share sensitive or embarrassing stories [42,43]. Users in OMHCs could interact with people who have similar mental health problems [20], seek, or provide social support [44,45]. To be specific, posts in OMHCs are considered as seeking social support, and comments in OMHCs are believed to provide social support [17,18].

As exchanging social support is one of the main motivations that drives users to join online communities [46,47], and online communities can develop successfully and sustainably only if enough active users provide social support [20], studies examined its relationship with user engagement and community development. Cunha et al. [22] did a causal analysis based on Mahalanobis Distance Matching and found that, in “loseit” weight loss community, users receiving more positive comments or upvotes on their initial post are more likely to return to the community in the future. Wohn and Lampe [21] conducted interviews with 30 users and found positive social support boosted subsequent feelings of pride, self-confidence, and status, and also motivated content contribution. Qiu et al. [23] found that 75% of users who received at least one reply from others will subsequently express positive sentiment in the future.

### 2.2. Online Empathy

In the process of providing social support or commenting in OMHCs, users could also express empathy to others they respond to. Empathy literally refers to “the power of understanding things outside ourselves” [48]. It is a multidimensional concept including both cognitive and affective components [49]. In the field of psychotherapy, empathy has long been recognized as an effective component in mental health counseling [50] and could result in positive outcomes in the therapeutic process [51]. Specifically, empathy involves the ability to understand the patient’s feelings, communicate that understanding and check its accuracy, and make use of that understanding in a helpful way [52]. Research also showed that empathy could improve intergroup relations [53,54] as well as reduce discrimination and prejudice to minority groups [55]. Decety and Fotopoulou [56] further proposed two mechanistic explanations to the positive effects of empathy, including social baseline theory and the free energy principle.

In addition to playing an important role in face-to-face interpersonal interactions, empathy is also found in online interactions [57]. Pfeil and Zaphiris [58] investigated empathy in an online community of older people and developed a coding scheme to analyze online empathy, including self-disclosure, light support, deep support, community-building, medical facts, and technical issues. Sharma et al. [25] further proposed a comprehensive structure of empathy in text-based, asynchronous, peer-to-peer support conversations, which consisted of three dimensions (reactions, interpretations, and explorations) and three levels in each dimension (no communication, weak communication, and strong communication).

Similar to offline empathy, research suggested that empathy was also associated with a positive outcome in OMHCs. Zhao et al. [24] found that empathy was positively related to individuals’ willingness to contribute support to others, which is crucial for the development of online communities. Sharma et al. [25] suggested that, after empathic interactions, more posters would follow the commenters. Considering the potential effects of online empathy in OMHCs, research examined ways to quantify empathy [25,26], and also proposed strategies to increase the empathy of responses [27,28].

In summary, although a rich body of work has shown the positive effects of social support on people’s mental health state and user contributions, few studies examined these effects from the perspective of online mental health communities, which has become a crucial resources for online mental health support [29]. In addition, prior research focused more on the general forms of social support such as the number of comments, while few studies further investigate the empathy level of online social support. Additionally, even though prior work has suggested the association between social support and empathy level [30], in the online context, we are not clear about how social support or empathy could affect or relate to users’ subsequent empathy expression. Therefore, our work seeks to address the research gap by focusing on OMHCs in Reddit and examining the impact of received social support and empathy on individuals’ subsequent behaviors. Specifically, this study aims to answer the following research questions:

- **RQ1:** Does received social support or empathy at the first post affect users’ probability of returning to online mental health communities?
- **RQ2:** Does received social support or empathy at the first post affect users’ subsequent empathy expression in online mental health communities?

## 3. Methods

To examine the effect of social support/empathy on user engagement, our main idea is to compare the treatment group and the control group that differed in received social support/empathy but were otherwise similar. Specifically, we collected accessible data from Reddit and proposed ways to measure social support and empathy. Then, in terms of social support, we matched users who received social support and those who did not receive social support, and compared their probability of returning to OMHCs and their subsequent expression of empathy. In terms of empathy, we matched users who received comments with high level of empathy and those who received comments with a low level of empathy, and did the same comparison. In addition, to test the stability of our results, we also tried other cutoff points to divide the treatment group and the control group. Finally, we conducted a preliminary content analysis to explore the difference between posts that received the most comments and those that received the least comments, and the difference between posts that received the most empathic comments and those that received the least empathic comments.

### 3.1. Data Collection

Reddit is a widely used online forum. Users can submit content to this forum in the form of posts, and they can also make comments on existing posts to continue the conversation. The posts in Reddit are organized by subject into a variety of communities called “subreddits”, which cover different topics such as sports, musics, news, and politics [29]. A lot of mental health related subreddits have been created where people could share mental health issues or offer social support to others struggling with mental problems. Prior research obtained a list of mental health related subreddits using snowball approach [43]. In this study, we focused on these 22 mental health related subreddits (also referred to as OMHCs)(The list of mental health related subreddit: r/depression, r/mentalhealth, r/Anger, r/ptsd, r/traumatoolbox, r/psychoticreddit, r/SuicideWatch, r/MMFB, r/getting_over_it, r/survivorsofabuse, r/alcoholism, r/BPD, r/rapecounseling, r/bipolarreddit, r/addiction, r/DPDR, r/hardshipmates, r/feelgood, r/StopSelfHarm, r/panicparty, r/socialanxiety, r/EatingDisorders).

**Figure 1:**
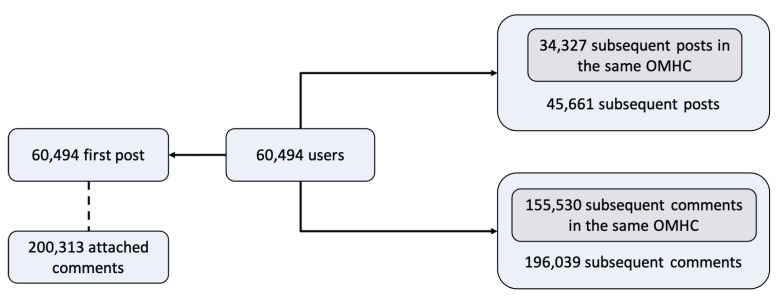
Statistics of the collected OMHC dataset. The “subsequent posts/comments in the same OMHC” means the posts/comments that users published subsequently in the same OMHC as their first post.

In order to examine the effect of social support and empathy at the first post, we selected users who satisfied two requirements: (1) has not posted or commented in any OMHCs from January 2018 to June 2018; and (2) has posted in OMHCs at least once from July 2018 to December 2018. The first requirement was set to make sure the user has not been involved in OMHCs for a relatively long period so his or her first post in the second requirement could be viewed as the first post in this study. We used Google’s BigQuery to obtain all users satisfying above requirements and collected their first post and attached comments. We also collected all their subsequent posts and comments in 22 OMHCs within a year after the first post. Since social support and empathy is an interpersonal behavior, this study only considered the comments that were made by users except the original poster. This provided us with 60,494 users, 60,494 corresponding first posts and 200,313 attached comments, 45,661 subsequent posts, and 196,039 subsequent comments (shown in Figure 1).

### 3.2. Preparation for Matching

Before creating the treatment group and control group, in this section, we first discuss the measures of two key variables in this study: social support and empathy. Then, for each variable, we prepare the candidates for the treatment group and the control group based on its value.

#### 3.2.1. Social Support

As comments are viewed as an important form of social support in online mental health communities [17,18], we measured the level of social support a user received by counting the number of comments they received. Therefore, we viewed users who received no comments on their first post as candidates for the control group and viewed users who received at least one comment on their first post as candidates for the treatment group. In this way, we obtained 46,256 candidates for the treatment group and 14,238 candidates for the control group.

#### 3.2.2. Online Empathy

As for online empathy, we first measured the empathy level of each comment, and then, for each post, we calculated the average empathy level of comments it received. Specifically, as empathy is a complex concept, we follow the framework of online empathy proposed by prior work [25], which demonstrated three main dimensions of empathy: emotional reactions (expressing emotions that commenters experience after reading the post), interpretations (communicating an understanding of experiences and feelings that commenters infer from the post), and explorations (exploring the experiences and feelings that are not included in the post to improve understanding). To measure the empathy level of comments in the above three dimensions, we built a RoBERTa-based classification model. This model was trained on a labeled dataset of Reddit created by Sharma et al. [25], which contains 3084 comments and their scores on three-point scale (0 to 2) in each dimension of empathy. Specifically, we split this labeled dataset into training, validation, and test sets in the ratio of 75:5:20, and the trained model’s test accuracy in three dimensions of empathy (emotional reactions, interpretations, and explorations) turned out to be 87.2%, 88.7%, and 92.9%, respectively.

**Figure 2:**
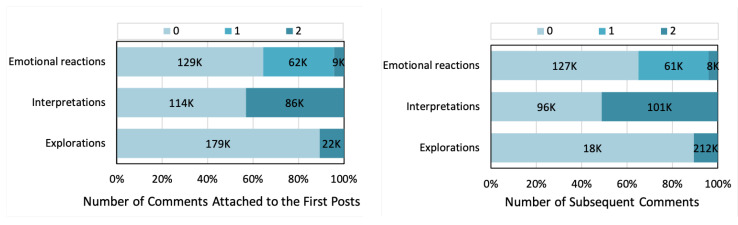
Empathy level of comments attached to users’ first posts (**left**) and users’ subsequent comments (**right**) in each dimension.

The basic statistics of empathy level of our data are shown in Figure 2. For each comment, we sum its scores in three empathy dimensions as its total empathy score. Then, for the post that received at least one comment, we calculated the average empathy score of comments it received. According to the median number (1.5), we divided the posts receiving at least one comment into candidates for the empathic treatment group (average empathy score of comments it received ≥1.5) and candidates for the non-empathic control group (average empathy score of comments it received <1.5). This finally provided us with 22,422 candidates for the treatment group and 23,834 candidates for the control group.

### 3.3. Propensity Score Matching

The simplest way to analyze the treatment effect is to subtract the outcome variable when someone does not receive treatment from that when someone receives treatment. However, we can not observe both results of being and not being treated in reality. Therefore, for each treatment factor in this study (social support and empathy), we matched similar pairs in which one received treatment while the other did not, then computed their difference to analyze the effect of treatment. The propensity score matching (PSM) [59] method matches users according to their probability of receiving treatment, and it could reduce the bias due to confounding variables [60]. In this study, we adopted PSM to match the treatment group and the control group and compared them to examine the treatment effect.

#### 3.3.1. Covariate Selection

Since users’ personal information such as demographics and images are not available in user profiles on Reddit, the selection of covariates in our research is based on the hypothesis that posts with similar content will receive similar comments [61]. In addition, since users’ engagement level in Reddit may also influence their subsequent behaviors such as return probability, we also consider relevant variables. Therefore, we chose the following covariates:

- Word Count: The number of words in the content (title + subtext) of posts.
- 20 LDA topic score: We generated a topical representation of the first post’s content by Latent Dirichlet Allocation (LDA) [62]. The parameters are set according to prior research [61].
- LIWC features: Linguistic Inquiry and Word Count (LIWC) [63] is a well-validated psycholinguistic lexicon which has been widely used in prior research [29,64]. In this study, we chose a set of 27 categories in LIWC that are suitable for mental health related content [65], e.g., anxiety, anger, and sadness.
- Empath features: We focused on 20 Empath [66] categories related to mental health to supplement LIWC features, e.g., nervousness, disappointment, and fear.
- VADER features: We used positive, negative, neutral, and compound scores generated by Valence Aware Dictionary for Sentiment Reasoning (VADER) [67] since it applies to social media data well.
- Dummy variables for OMHCs: Considering the potential difference of 22 OMHCs such as activity level and community rules, we created 21 dummy variables for these OMHCs.
- User engagement level: We measured the level of user engagement on Reddit by calculating the total number of posts and comments they published on Reddit, and the average word count of these posts and comments in a period of six months before the user’s first post.

We then adopted a logistic regression with LASSO (Least Absolute Shrinkage and Selection Operator) to further select variables for PSM from all above features. LASSO is a regression analysis method that performs both variable selection and regularizations. We computed the mean AUC (Area Under the receiver operating characteristic Curve) over 10-fold cross validation setting. For the first treatment factor (social support), we finally obtained a model including 19 variables, in which AUC is 0.66. For the second treatment factor (empathy), we finally obtained a model including 13 variables, in which AUC is 0.56.

#### 3.3.2. Matching and Validation

We conducted propensity score matching to remove the relationship between the covariates and supposed causal variable. Based on selected variables, we conducted one-to-one nearest neighbor matching with a caliper of 0.01. If there was more than one matched user to a certain user, we selected the pair with the smallest Mahalanobis distance. For the first treatment factor (social support), we finally obtained 13,769 pairs of users; for the second treatment factor (empathy), we obtained 20,464 pairs of users.

We checked the absolute standardized mean difference (ASMD) of covariates before and after matching, and the results showed that the ASMD of all covariates after matching was less than 0.1, which meant that the treatment group and control group were well balanced [68]. Therefore, we could compare the treatment group and control group to investigate the treatment effects.

### 3.4. Stability Analysis

In the above analysis of the effect of social support, we divided users into the treatment group and the control group according to whether they received any comment and compared these two groups. To test the stability of our results, we also tried other sets of the cutoff point and used the same method to see if there is always a difference between receiving more comments and fewer comments. Specifically, we set the cutoff point *k* to 0,1,...,4, respectively. In each case, similar to the analysis mentioned in the above sections, we divided all users into the treatment group (received more than *k* comments at the first post) and control group (received no more than *k* comments at the first post), and conducted propensity score matching and checked the balance of these two groups. Finally, we compared return probability and expression of empathy of users who received more comments with those who received fewer comments.

In terms of empathy, we also conducted a similar stability analysis. In addition to dividing users into treatment group and control group according to the median value of empathy level that users received, we also tried different treatment cutoff to see if there is always a difference between receiving more empathic comments and less empathic comments. Specifically, we set the cutoff point *k* to 0,0.5,1,1.5,2, respectively, and accordingly divided all users into the treatment group (average empathy score of received comments at the first post >k) and control group (average empathy score of received comments at the first post ≤k). In each case, we conducted propensity score matching and checked the balance between these two groups. Finally, we compared return probability and expression of empathy of users who received more empathic comments with those who received less empathic comments.

### 3.5. Content Analysis

After examining the effect of received social support and empathy, we explored the difference between (1) posts that received most comments and posts received that fewest comments, and the difference between (2) posts that received the most empathic comments and posts that received the least empathic comments. This preliminary content analysis would provide us with insights into what kind of posts would receive more comments or more empathic comments, and therefore inform the design of social media to better support people.

Specifically, for our four corpora—posts with no comment (*n* = 14,238), posts with most comments (*n* = 14,238), posts with non-empathic comments (*n* = 4958), and posts with most empathic comments (*n* = 4958), we first stemmed the words in each dataset. To avoid the overly bias for operational or function words [69], we calculated TF-IDF (term frequency–inverse document frequency) of each token to evaluate their importance in the corpus instead of directly counting the frequency of each token. Then, we compared the tokens with the highest TF-IDF weight in each corpus to explore their difference in the content of posts.

## 4. Results

In this section, we compared the outcome variables of two pairs of matched groups to investigate the effect of social support the empathy. Prior research has shown the difference between posts and comments in OMHCs: that posts are mainly published to seek support while comments are used to provide support [17,18], so we examined the user behaviors about posts and comments separately. We also presented the results of stability analysis and preliminary content analysis.

### 4.1. Return Probability(RQ1)

#### 4.1.1. Comment vs. No Comment

For the “comment group” that received at least one comment on the first post and “no comment group” that did not receive any comment on the first post, we first compared their probabilities of returning to the OMHCs to post or comment. Specifically, we considered the probabilities of the following four activities: (1) returning to post in the same OMHC as the first post, (2) returning to post in any OMHC, (3) returning to comment in the same OMHC as the first post, and (4) returning to comment in any OMHC. As Table 1 shows, all four of these return probabilities of the “comment group” are significantly higher than the “no comment group”.

The probability of returning to post in the same OMHC and in any OMHC in the “comment group” is 17.7% and 12.8% relatively higher than that in the “no comment group”, respectively, and the differences are both significant. Since we have controlled the differences between the two groups in terms of sentiment, topics, and expression way of their first posts, the results suggest that the higher post return probability of the “comment group” is possibly caused by receiving comments on their first post. Prior research has shown that users might post in OMHCs to seek social support [17,18], and we also randomly selected 1000 posts from our dataset and manually coded them to check this claim. The results suggest that 94.7% of 927 unremoved posts include users sharing their personal experience and seek support. Therefore, our results in this section suggest that social support increases the probability of subsequent self-disclosure and support seeking behaviors in the OMHCs.

The difference in the probability of returning to OMHCs to comment between the two groups is also significant. Users in the “comment group” were 15.8% relatively more likely to comment to others in the same OMHC, and 15.3% relatively more likely to comment to others in any OMHC after the first post. These results show that social support is “contagious”, which means receiving social support (comment) on the first post significantly increases the probability of providing others with social support subsequently.

#### 4.1.2. Empathic Comment vs. Non-Empathic Comment

For users who received at least one comment, we further explored the effect of average empathy level of their received comments. As Table 2 shows, users who received more empathic comments on the first posts were more likely to return to post in OMHCs, while the difference is not significant. Regarding the commenting behavior, the return probability of the “empathic comment group” is 5.2% relatively higher than that of the “non-empathic comment group” both in the same OMHC as the first post and in any OMHC, and the differences are significant. These results suggest that a higher level of empathy a user received is effective in increasing the probability of returning to OMHCs to publish subsequent posts or comments.

### 4.2. Expression of Empathy (RQ2)

To better understand the impact of social support and empathy on users’ subsequent expression of empathy, we also conducted propensity score matching and compared subsequent expression of empathy between users in the treatment group and the balanced control group. Specifically, we focused on the average empathy score of users’ subsequent *k* (*k* = 1, 2,..., 10) comments in OMHCs and in the same OMHC as their first posts, respectively.

#### 4.2.1. Comment vs. No Comment

First, we compared the expression of empathy between users who received at least one comment and those who did not receive social support. The results (Table 3) show no significant difference between two groups in subsequent expressions of empathy. This indicates that, even though whether users received social support or not was closely related to their probability of returning to online mental health communities, it did not affect the level of empathy that users subsequently expressed to others.

#### 4.2.2. Empathic Comment or Non-Empathic Comment

Then, we explored the impact of average empathy level of comments users received on their further expression of empathy. As Table 4 shows, when we considered a certain number of subsequent comments, “empathic group” would also publish more empathic comments to others, and the difference is significant except for the case where *k* is 1. These results show that, similar to the probability of providing social support, the expression of empathy could also propagate in OMHCs.

### 4.3. Stability Analysis

To test the stability of above results, in this section, we present the results based on different sets of the cutoff point.

#### 4.3.1. Return Probability

**Figure 3:**
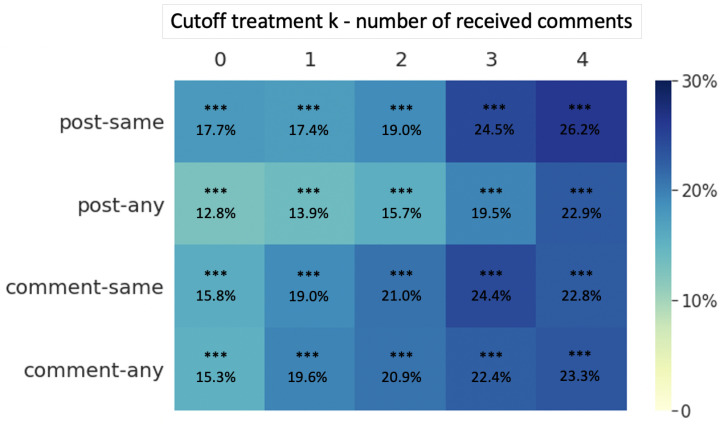
Effect size of the difference in return probability between more comments group and fewer comments group in different treatment definitions (more comments group: numberofreceivedcomments>k, fewer comments group: numberofreceivedcomments≤k. *** *p* < 0.001.

For the comparison between users who received more comments and those who received fewer comments, the results (Figure 3) show that, no matter which cutoff point is chosen, receiving more comments on the first posts always leads to a higher subsequent return probability. However, unlike previous research [61], our results did not show the effect of “diminishing returns”; instead, the treatment effect is even greater when the cutoff point is set greater.

**Figure 4:**
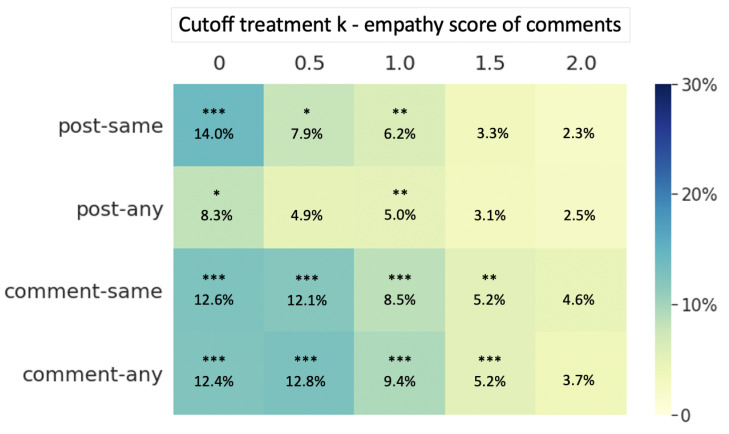
Effect size of the difference in return probability between more empathic group and less empathic group in different treatment definitions (more empathic group: averageempathyscoreofreceivedcomments>k, less empathic group: averageempathyscoreofreceivedcomments≤k). * *p* < 0.05, ** *p* < 0.01, *** *p* < 0.001.

For the comparison between users who received more empathic comments and those who received less empathic comments, as Figure 4 shows, a more empathic group always has a higher return probability, regardless of the cutoff point chosen. The results also suggest the “diminishing effect” of empathy: as the cutoff point of the empath score is larger, the difference between the more empathic group and less empathic group is smaller. In other words, when the average empathy level of comments a user received is relatively low, the increase in that empathy level is more effective in increasing users’ return probability.

#### 4.3.2. Expression of Empathy

For the comparison between users who received more comments and those who received fewer comments, the results show that, no matter which treatment cutoff is chosen, there is no significant difference between users who received more comments at their first posts and those who received fewer comments at their first posts.

For the comparison between users who received more empathic comments and those who received less empathic comments, the results show that, when we divide the empathic group and non-empathic group according to several moderate cutoff points (0.5, 1, 1.5), the average empathy score of users’ subsequent comments in the more empathic group is significantly higher than the less empathic group. In addition, in these cases, we also find the “diminishing effect”, which means that, when the cutoff point of empathy is set higher, the effect size is smaller. That is, when the empathy level of received comments is relatively low, increasing this level would be more effective in increasing users’ subsequent expression of empathy. The reason why two groups have no significant difference in subsequent expression of empathy when *k* is set to 0 or 2 might be the fact that, when the cutoff point is too high or too low, there are too few matched pairs left, which makes it difficult to obtain reasonable results.

### 4.4. Content Analysis

In this section, we show the results of a preliminary content analysis of four corpora of posts that received a different number of comments or comments with different levels of empathy. For each corpus, we present the top 20 tokens with the highest TF-IDF weight.

#### Comment vs. No-Comment

As Table 5 suggests, most of the top 20 words and their popularity in “most comments group” and “no comment group” are similar, such as “want”, “depress”, “know”, “life”, “friend”, and “help”. These words show that self-disclosure about difficulties of life are common in OMHCs, and therefore they appear frequently in both corpora. We also observed the difference in the use of some tokens between “most comment group” and “no comment group”. For example, the TF-IDF weight of “suicid” and “drink” in “comment group” are higher than those in “no comment group”. This shows that, when users are in difficult situations and mention emotional activities which might be harmful to themselves, others might be more likely to provide them with social support. Though we only did a preliminary content analysis of the tokens in “most comments group” and “no comment group” and could not draw causal conclusions, the results provide insights into future research that could thoroughly investigate which kinds of posts will receive more social support in OMHCs.

### 4.5. Empathic vs. Non-Empathic

We also compared the most popular tokens in “most empathic group” and “non-empathic group”. The results suggest (Table 6) that some tokens related to emotions obtained higher TF-IDF weight in “most empathic group” than in “non-empathic group”, including positive emotions such as “love” and negative emotions such as “hate”. Prior work suggests that empathic responses often mimic the emotion of the original user or speaker to a certain degree [27], and our results imply that the emotional expression in a post might be related to the empathy level of its comments. Additionally, similar to the comparison between “comment group” and “no comment group”, we also found the higher weight of “suicid” in “most empathic group” than in “non-empathic group”, which means the self-disclosure regarding suicide might not only attract more comments, but also be associated with a higher empathy level of received comments.

## 5. Discussion

### 5.1. Impact of Receiving Social Support

Our results indicate that the users who received at least one comment from others would be more likely to return to OMHCs to post or comment. This is aligned with existing studies about the relationship between feedback and content contribution [70,71] but provides new insights into the area of social support. Since we focused on OMHCs, our results show the significant effect of social support on users with mental health concerns. Additionally, as prior research showed that posters in OMHCs act as support seekers and commenters in OMHCs act as support providers [17,18], our results suggest that users who received social support at the first post in OMHCs would be more likely to continue to disclose themselves and seek help, and offer others support in the future. In addition to dividing users into “comment group” and “no comment group’, we also set the cutoff point of the number of comments to different values and checked the treatment effect in each case. The results do not show the “diminishing effects” as prior research on an online weight-loss community suggested [61]. Instead, the difference between “more comments group” and “fewer comments group” would be even greater when the cutoff point was set greater. However, as for the subsequent expression of empathy to others, we did not find a significant difference between the “comment group” and the “no comment group”. In summary, this study suggests that receiving social support might lead to more active subsequent user engagement, including the probability of social support provision and self-disclosure behaviors, but it did not have a significant effect on users’ subsequent empathy expression in those behaviors.

### 5.2. Impact of Empathy Level of Comment

This study further examined the relationship between the empathy level of received comments and users’ subsequent online behaviors. As for the return probability, this study suggests that the users in the “empathic group” that received comments with a higher empathy level would be more likely to provide social support for others. This result is aligned with research that showed the positive relationship between empathy and user engagement such as content contribution [24] and interpersonal interactions [25], while also contributing to the big picture of online empathy since we consider a different online behavior—providing others with social support. We also tried different cutoff points of the average empathy score to divide users into “empathic group” and “non-empathic group”. When increasing the cutoff point, we observed the diminishing effect size. This means that, when a post received comments with a relatively low average empathy level, it would be more effective to improve the empathy level of comments for increasing the users’ subsequent engagement.

In addition, we examined the propagation of empathy in OMHCs by analyzing the effect of empathy level of received comments on users’ subsequent expression of empathy. Our findings show that users who received more empathic comments would publish significantly more empathic comments on others’ posts. Our findings about online empathy are similar to the “emotional contagion” phenomenon, which means that one person’s emotions and related behaviors may trigger similar emotions and behaviors of other people [72]. This study shows that online empathy could also be contagious in OMHCs, and therefore contributes to the atmosphere of OMHCs. All these results provide insights into the design of OMHCs to develop successfully and better support their users.

### 5.3. Implications for Design

This study informs readers that, in online mental health communities, initially received social support and empathy may have a significant impact on individuals’ future behaviors. This may result in chain reactions: received social support or empathy may affect an individual, and encourage him or her to provide others with social support; therefore, more users will be affected, and the number of affected users may continue to increase when this process repeats. Therefore, it would be beneficial for the whole community if each user could receive warm social support on the first post. To this end, platforms could identify and call attention to posts that have not received any responses, e.g., creating a special “unanswered posts” section and providing higher incentives for users who reply to these posts. In addition, setting up a response reminding system and timely informing users of their received social support may further strengthen the chain effect of social support. In addition, the OMHCs could appropriately introduce robots to automatically add more empathic comments or guide users to improve the empathy level of comments under certain posts (e.g., those expressing a suicidal tendency) to better support users and keep a healthy and empathic community atmosphere.

## 6. Conclusions

This study examined the effect of social support and empathy in the form of comments in online mental health communities. We focused on the users in 22 subreddits, and matched and compared users (1) who received social support with those who did not receive social support, and users (2) who received more empathic social support with those who received less empathic social support. Our results showed that social support and empathy are contagious. That is, users who received social support on the first post would be more likely to post again and provide support for others; in addition, users who received more empathic support would subsequently provide more empathic social support for others. We also did a preliminary content analysis on posts that received different numbers of comments and comments with different levels of empathy. Moreover, this study provides insights into how online mental health communities can serve as a better place for people to spread social support.

## 7. Limitations and Future Work

A current limitation of our study was the incomplete selection of mental health related subreddits. Although this study included main OMHCs on Reddit which were demonstrated in prior research, we noticed that there are some other OMHCs with fewer members that also existed, which might cause bias in our results such as the probability to return to post or comment on any OMHC. In addition, we only took into account the effect of social support and empathy on users’ first posts. This is because it is difficult to separate the impact of social support or empathy that a user has received on each post among their multiple posts. It is worth exploring further how to measure the marginal effect of social support or empathy on each post separately. Additionally, though this study examined the effect of social support and empathy on users’ subsequent posting or commenting behaviors, a more detailed analysis is needed to examine the specific reason why users posted. Future work could analyze the content of these posts to examine whether users returned to OMHCs to seek help on new issues or they were not satisfied with the support they had received and tried to seek help again.

The following abbreviations are used in this manuscript:

## Figures and Tables

**Table 1:** Comparing the return probability of “comment group” and “no comment group”. EATE *(estimated average treatment effect)* =(Y(treatment)−Y(control))/Y(control),Y=outcomevariable. *** *p* < 0.001.

Variable	Comment Group	No Comment Group	EATE	Chi-Square
Post in the same OMHC	22.9%	19.5%	17.7%	48.833 ***
Post in any OMHC	26.9%	23.8%	12.8%	33.692 ***
Comment in the same OMHC	29.2%	25.2%	15.8%	55.096 ***
Comment in any OMHC	34.5%	29.9%	15.3%	65.996 ***

**Table 2:** Comparing the return probability of “empathic comment group” and “non-empathic comment group”. ** *p* < 0.01, *** *p* < 0.001.

Variable	Empathic Group	Non-Empathic Group	EATE	Chi-Square
Post in the same OMHC	22.4%	21.6%	3.3%	2.994
Post in any OMHC	27.0%	26.2%	3.1%	3.368
Comment in the same OMHC	29.2%	27.7%	5.2%	10.378 **
Comment any OMHC	35.1%	33.4%	5.2%	13.444 ***

**Table 3:** The difference in the empathy level of users’ subsequent *k* comments between “comment group”(left) and “no comment group”(right), and their respective values.

	k	1	2	3	4	5
Variable						
Comment in the same OMHC		−0.2% (1.764,1.767)	−0.2% (1.745,1.749)	0.4% (1.746,1.739)	0.8% (1.746,1.732)	1.8% (1.747,1.716)
Comment in any OMHC		−1.4% (1.725,1.750)	−0.6% (1.704,1.715)	1.1% (1.702,1.683)	0.1% (1.688,1.687)	0.0% (1.678,1.678)
		**6**	**7**	**8**	**9**	**10**
Comment in the same OMHC		1.5% (1.737,1.712)	1.1% (1.719,1.700)	1.9% (1.708,1.676)	1.8% (1.701,1.670)	2.5% (1.696,1.654)
Comment in any OMHC		1.0% (1.681,1.665)	2.0% (1.681,1.647)	1.6% (1.668,1.642)	0.8% (1.663,1.649)	1.4% (1.662,1.640)

**Table 4:** The difference in the empathy score of users’ subsequent *k* comments between “empathic comment group” (left) and “non-empathic comment group” (right), and their respective values. * *p* < 0.05, ** *p* < 0.01, *** *p* < 0.001.

	k	1	2	3	4	5
Variable						
Comment in the same OMHC		2.1% (1.812,1.775)	2.6% * (1.797,1.752)	3.3% ** (1.788,1.732)	3.6% ** (1.780,1.719)	3.5% ** (1.775,1.714)
Comment in any OMHC		1.5% (1.778,1.751)	2.7% ** (1.772,1.726)	2.6% ** (1.753,1.708)	3.0% ** (1.741,1.691)	3.8% *** (1.735,1.672)
		**6**	**7**	**8**	**9**	**10**
Comment in the same OMHC		3.0% * (1.765,1.713)	2.7% * (1.754,1.709)	3.9% ** (1.758,1.692)	5.1% *** (1.768,1.683)	5.2% *** (1.756,1.669)
Comment in any OMHC		3.6% *** (1.726,1.666)	3.6% *** (1.718,1.658)	3.6% *** (1.713,1.653)	3.6% *** (1.714,1.655)	3.7% *** (1.716,1.654)

**Table 5:** Top 20 tokens with highest TF-IDF weight in “most comment group” and “no comments group”.

Most Comments Group		No Comment Group	
Token	Weight	Token	Weight
want	0.0255	want	0.0262
know	0.0234	depress	0.0259
depress	0.0229	know	0.0239
friend	0.0224	help	0.0236
peopl	0.0220	friend	0.0234
life	0.0217	life	0.0222
help	0.0214	think	0.0212
think	0.0210	peopl	0.0206
time	0.0203	time	0.0206
year	0.0197	year	0.0198
go	0.0197	talk	0.0193
talk	0.0193	go	0.0190
tell	0.0185	thing	0.0187
drink	0.0180	work	0.0179
fuck	0.0180	need	0.0169
thing	0.0179	tell	0.0164
work	0.0164	fuck	0.0159
love	0.0162	start	0.0156
suicid	0.0162	love	0.0155
need	0.0161	live	0.0153

**Table 6:** Top 20 tokens with highest TF-IDF weight in “most empathic group” and “non-empathic group”.

Most Empathic Group		Non-Empathic Group	
Token	Weight	Token	Weight
friend	0.0242	depress	0.0259
depress	0.0225	want	0.0257
peopl	0.0220	help	0.0242
help	0.0219	know	0.0242
year	0.0218	life	0.0231
talk	0.0205	friend	0.0228
go	0.0204	think	0.0220
thing	0.0198	peopl	0.0210
fuck	0.0198	time	0.0206
tell	0.0195	year	0.0198
work	0.0193	go	0.0192
anymor	0.0190	talk	0.0189
love	0.0187	thing	0.0186
school	0.0180	work	0.0181
live	0.0180	need	0.0176
start	0.0177	tell	0.0162
tri	0.0177	love	0.0159
need	0.0176	start	0.0159
hate	0.0161	fuck	0.0158
suicid	0.0161	live	0.0155

## Data Availability

Not applicable.
